# Optimizing antiviral therapy for COVID-19 with learned pathogenic model

**DOI:** 10.1038/s41598-022-10929-y

**Published:** 2022-04-27

**Authors:** Abhishek Dutta

**Affiliations:** Department of Electrical & Computer Engineering, Storrs, 06269 USA

**Keywords:** Viral infection, Computational models, Systems analysis, Biomedical engineering

## Abstract

COVID-19 together with variants have caused an unprecedented amount of mental and economic turmoil with ever increasing fatality and no proven therapies in sight. The healthcare industry is racing to find a cure with multitude of clinical trials underway to access the efficacy of repurposed antivirals, however the much needed insights into the dynamics of pathogenesis of SARS-CoV-2 and corresponding pharmacology of antivirals are lacking. This paper introduces systematic pathological model learning of COVID-19 dynamics followed by derivative free optimization based multi objective drug rescheduling. The pathological model learnt from clinical data of severe COVID-19 patients treated with remdesivir could additionally predict immune T cells response and resulted in a dramatic reduction in remdesivir dose and schedule leading to lower toxicities, however maintaining a high virological efficacy.

## Introduction

COVID-19 has been relentless in finding ways to infect people of all age groups and demographics worldwide. This has led to catastrophic consequences in terms of human lives lost, job loss and mental fatigue. The RNA vaccines amongst others have certainly helped to counter COVID-19 to some extent but its emerging more transmissible and lethal variants including the delta variant has led to rapid rise in infections and hospitalisations across the globe^1^. This necessitates a systematic multidisciplinary approach to antiviral therapeutics that are not only efficacious in inhibiting the pathogenesis of SARS-CoV-2 but also are less toxic. Though systems engineering perspectives are uncommon in pathological modeling and therapeutic control, in this case healthcare system could greatly benefit from one^2^.

Coronaviruses can be found in different species of animals and can evolve and infect humans. Previous outbreaks to COVID-19 were the Severe Acute Respiratory Syndrome (SARS-CoV) of 2003 and later, in 2012, the Middle East respiratory syndrome (MERS-CoV). Metagenomics studies previous to the COVID-19 outbreak envisaged the possibility of future 33 threats due to the identification of several sequences closely related SARS-like viruses circulating in the wild^3^. To date the SARS-CoV-2 has infected 500 million and caused 6 million fatalities across the world.

Most patients who become critically ill following infection with SARS-CoV-2, the causative agent of COVID-19, develop acute respiratory distress syndrome (ARDS)^4^. The deterioration of lung function and then leading to possible multiple organ failure can be attributed to a positive feedback loop of the inflammatory immune response aided by the cytokine storm. Currently, COVID-19 management is mostly limited to palliative and symptomatic treatment. A multitude of trials involving candidate antiviral drugs and other anti-inflammatory agents are underway in a race to develop more effective therapies^5^. These drugs inhibit viral membrane fusion and endocytosis, viral entry (via the angiotensin-converting enzyme 2 [ACE2] receptor and transmembrane serine protease 2 [TMPRSS2]), or the activity of the RNA-dependent RNA polymerase and the SARS-CoV-2 3-chymotrypsin-like protease (3CLpro)^6^.

The COVID-19 pandemic is unlike any other recent disease outbreak for several reasons including a surprisingly high basic reproduction number and a large percentage of people who contract the disease progress to viral pneumonia overwhelming the hospitals^7^. Although therapeutic agents have the potential to be useful tools in ameliorating symptoms and reducing mortality in patients, the development of effective drugs against SARS-CoV-2 has multiple challenges that require careful investigation. For instance, the FDA approved antiviral therapy remdesivir, can have potential adverse drug events including hypersensitivity reactions and increase in levels of liver enzyme^8^. Therefore, a comprehensive analysis, grounded in clinical data from hospitalizations due to the novel coronavirus need to be carried out to analyze the fundamental factors of the treatment in terms of timing and duration of initiation, drug potency, short and long-term toxicities to guide the rational identification and re purposing antiviral therapies^9^.

Virological modeling and pharmaco kinetic/dynamic study of antiviral drugs have been performed on pandemics such as HIV and its progression to AIDS^10^ and strains of the Influenza virus^11^ amongst others. Subsequently, the potential for controlled treatment scheduling has been shown for HIV^12,13^. However, most of the effort on mathematical modeling and analysis of COVID-19 has been on the epidemiological level^14–16^ with limited work on the viral kinetics of SARS-CoV-2^17^. Yet, there is no work connecting viral and immune system interactions with the systems pharmacology and toxicology of antiviral therapeutic drugs. In this paper, a virology-immunology dynamic model of COVID-19 is learnt for a prescribed antiviral therapy remdesivir to explain the clinically observed viral load amongst severely infected patients. Next, for the first time, the viral load is controlled with an optimized remdesivir therapy in terms of its dosage and scheduling by derivative free global optimization. A toxicology model further demonstrates the efficacy of the new regimen.

## Results

### Pathology pharmacology model

Coronavirus enters target cells through an endosomal pathway. S protein first binds to cellular receptor ACE2, and ACE2-virus complex is then translocated to endosomes, where S protein is cleaved by endosomal acid proteases and viral genome is released and translated into viral replicase polyproteins, which are then cleaved into small products by viral proteinases. Viral nucleocapsids are assembled from genomic RNA and N protein in cytoplasm, followed by budding into lumen of the Endoplasmic Reticulum Golgi Intermediate Compartment. Virions are then released from cell through exocytosis. These processes can be inhibited at multiple levels by several existing drugs such as Choloroquine is shown to interfere with terminal glycosylation of ACE2 and Camostat, Nafamostat are both clinically proven inhibitors of TMPRSS2. During viral proteolysis, Lopinavir and Ritonavir, are known to inhibit the activity of 3CLpro and is approved for the treatment of HIV/AIDS. During viral replication: remdesivir, an adenosine analog, developed to combat other viruses such as Ebola, arrests RNA synthesis. During host cytokine response, monoclonal antibodies (mAbs) against the IL-6 receptor, Sarilumab, Tocilizumab, are emerging as possible treatments for COVID-19 patients.

**Figure 1:**
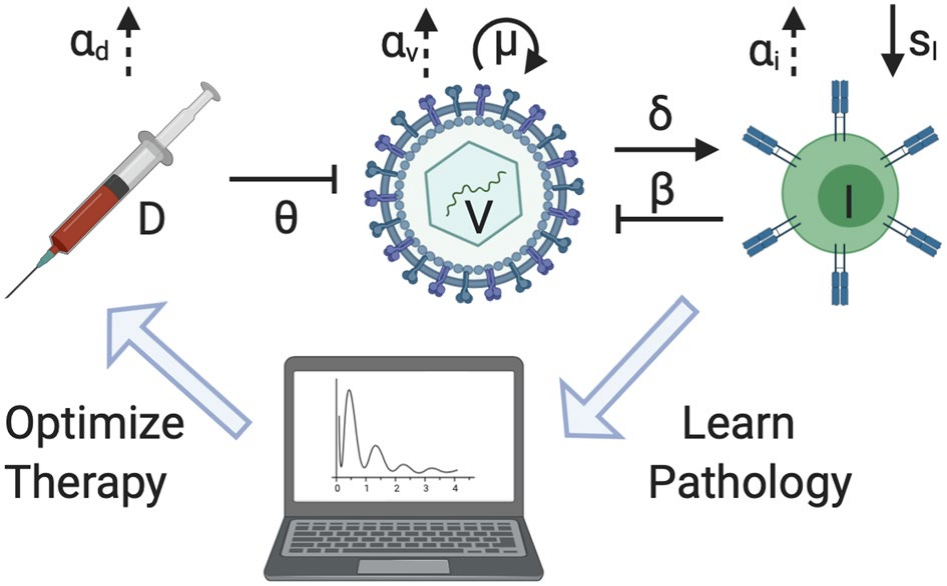
The pathology pharmacology model of COVID-19 is constructed with inhibition and solid/dotted arrows representing proliferation/clearance. Subsequently, the model parameters can be learnt for optimized therapy.

The target cell limited model has been widely used to generate the dynamics between uninfected and infected cells through viral loading, proliferation and clearance in infectious diseases such as HIV^18^ and more recently SARS-CoV-2^17^. However, in the case of COVID-19, the immune response dynamics does have an even more significant role to play^19^. In particular, the role of T cells in the resolution or exacerbation of COVID-19, as well as their potential to provide long-term protection from reinfection with SARS-CoV-2 has been highlighted^20^. This necessitates the consideration of a dynamic model that includes both the viral dynamics *V* as well as the immune T cell dynamics *I* of the disease within the patient body^21^. The interaction model can then be formulated as:

1 $$\begin{aligned}&\dot{V} = (1-\theta )\mu V(1-\frac{V}{\sigma _v})-\beta VI-\alpha _vV \end{aligned}$$

2 $$\begin{aligned}&\dot{I} = s_I+\delta I(\frac{V}{V+\sigma _i})-\alpha _iI \end{aligned}$$

where the viral replication is modeled with a logistic function with maximum carrying capacity $\sigma _v$. The virus replicated with a rate of $\mu$ and gets cleared with a rate of $\alpha _v$. The virus gets eliminated by the immune response at a rate $\beta$ through the coupling term $\beta VI$. The immune T cells supply $s_I$ is determined by homeostasis in conjunction with the rate of clearance $\alpha _i$. The immune cells proliferate with a rate $\delta$ upon activation by the virus through various signalling pathways represented by Michaelis Menton function with half saturation constant $\sigma _i$^22^. The parameter $\theta$ represents the efficacy of antiviral therapeutic intervention.

Pharmacokinetic/pharmacodynamic (PK/PD) models have been employed at all stages of drug discovery and application by pharmaceutical companies^23^. PK and PD interactions describe the dynamic of drug in body and body’s response to the drug respectively as follows

3 $$\begin{aligned}&\dot{D}=-\alpha _dD\;\; \tau _k<t<\tau _{k+1},\;\; k=1,2\ldots N \end{aligned}$$

4 $$\begin{aligned}&\theta =\frac{D}{D+EC_{50}} \end{aligned}$$

where PKs are modeled by (3) and describe instantaneous and complete absorption of the drug. *D* is the amount of the drug in the blood and $\alpha _d$ is its elimination rate. The drug is administered at every $\tau _k$ with $k=1\ldots N$ as the schedule. PDs are modeled by (4) determining the efficacy of the antiviral treatment and $EC_{50}$ is the drug concentration that leads to a $50\%$ effectiveness. The structure of closed-loop controlled therapy based on learnt pathology immunology model can be seen in Fig. 1.

### Learning COVID-19 pathology

The antiviral therapeutic potency and consequential parameters like $EC_{50}$ are usually measured through in vitro assays that being based on immortalized non-human cell lines do not as such reflect the true pathogenic complexities of the novel coronavirus line of RNA virus^9^. Consequently, the identification of the pathological and pharmacological parameters is an important step to help in interpreting experimental data from humans. Taking into account immune T cell homeostasis of $s_I=\alpha _iI_0$, where $I_0$ is the initial immune T cell count, $s_I,\alpha _i$ can be analytically computed and from the half life of an antiviral drug, $\alpha _d$ can be deduced. The rest of the pathological parameters $\mu ,\beta ,\sigma _v,\alpha _v,\delta ,\sigma _i$ have to be identified from patient data. Note also that the drug response constant $EC_{50}$ though available for cell cultures is not the same for humans and so should be optimized. Finally, the viral load measured by Reverse Transcription Polymerase Chain Reaction (RTPCR) in hospitals often corresponds to a period well after incubation $\epsilon$ when symptoms are rather pronounced, hence $\epsilon$ must be identified as well. The optimal parameters are the solution to the following minimization problem

5 $$\begin{aligned} \min _{\mu ,\beta ,\sigma _v,\alpha _v,\delta ,\sigma _i,EC_{50},\epsilon }&\;\;\Vert \mathbf {V}-\mathbf {V}_{PCR}\Vert ^2 \nonumber \\ \textit{where}\qquad&\dot{V} = \frac{EC_{50}}{D+EC_{50}}\mu V(1-\frac{V}{\sigma _v})-\beta VI-\alpha _vV \nonumber \\&\dot{I} = s_I+\delta I(\frac{V}{V+\sigma _i})-\alpha _iI \nonumber \\&\dot{D}=-\alpha _dD\;\; \tau _k<t<\tau _{k+1},\;\; k=1,2\ldots N \end{aligned}$$

where the cost function represents the squared norm $\Vert \cdot \Vert$ of the error between the vector of viral titre measured by PCR $\mathbf {V}_{PCR}$ and the vector of model predictions $\mathbf {V}$ beyond the incubation period of $\epsilon$ responding to an FDA approved drug schedule.

Remdesivir (also GS-5734) is a monophosphoramidate prodrug of an adenosine analogue that has a broad antiviral activity including coronaviruses, pneumoviruses, paramyxoviruses and filoviruses.

In vitro, remdesivir inhibits all animal and human coronaviruses tested to date, including SARS-CoV-2, and has been clinically efficacious in animal models of Middle East respiratory syndrome (MERS)-CoV and SARS-CoV-1 infections^24^. Remdesivir is a formidable inhibitor of SARS-CoV-2 replication in bronchial airway epithelial and nasal cells in humans.

A multi centre clinical trial of remdesivir in adults with severe COVID-19 was performed at ten hospitals in Hubei, China with a presentation of the resulting clinical data^25^. Eligible patients were non-pregnant women and men with COVID-19 who were aged at least 18 years and were RT-PCR positive for SARS-CoV-2, had a ratio of arterial oxygen partial pressure to fractional inspired oxygen of 300 mm Hg or less or oxygen saturation of $94\%$ or lower on room air, had pneumonia confirmed by chest imaging and were within 12 days of symptom onset.

Target patients received intravenous remdesivir (200 mg on day 1 followed by 100 mg on days 2–10 in single daily infusions) for a total of 10 days (provided by Gilead Sciences, USA). Oropharyngeal or nasopharyngeal swabs, faecal or anal swab specimens and expectorated sputa as available were collected on days 1, 3, 5, 7, 10, 14, 21, and 28 for viral RNA quantification and detection^25^, forming the basis for the pathological model learning and optimized antiviral therapy analysis.

The viral load by quantitative PCR on the upper respiratory tract specimens were collected on days 1, 3, 5, 7, 10, 14, 21, and 28 and systemically tabulated^25^. Though, the pharmacokinetics of remdesivir in these severely ill patients, and particularly the concentrations of the active nucleotide metabolite (GS-441524) triphosphate in respiratory tract cells of treated patients, were unknown. This paper contributes to the understanding of the dynamic interaction of remdesivir and the pathogenesis of SARS-Cov-2 in severe COVID-19 patients.

**Figure 2:**
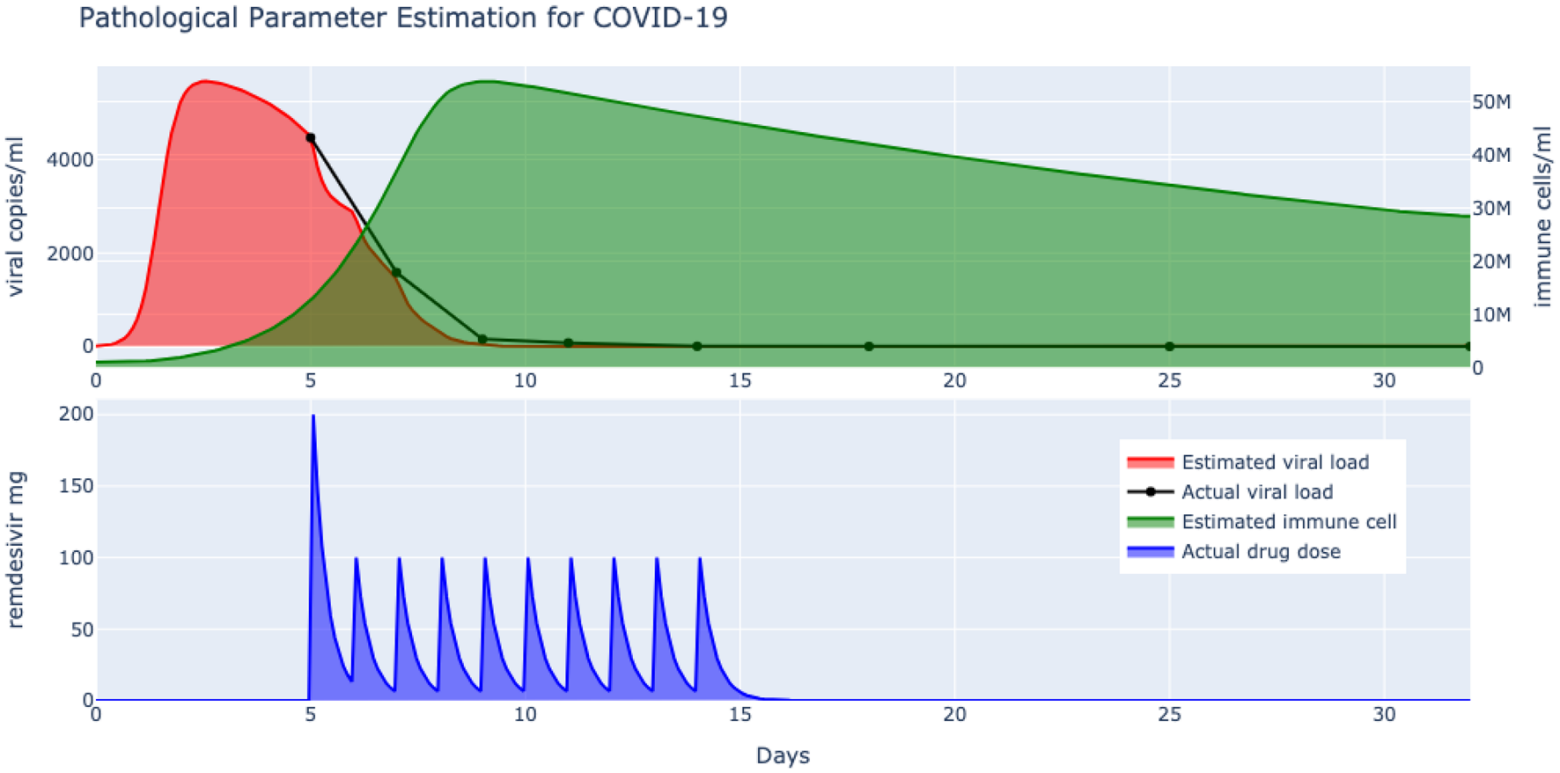
Parameter learning of remdesivir targeted SARS-CoV-2 model by derivative free optimization shows match with reported deceleration in viral load. Subsequently, the immune system response is also predicted.

The number of immune T cells was initialized to $10^6$ and its rate of clearance follows from the half life of T cells which is about 30 days^26^. The initial viral titre is below the 100 copies/ml mark which is the limit of detection and remdesivir is only administered after the incubation period in this study (200 mg on day 1 followed by 100 mg on days 2–10 in single daily infusions). All the remaining pathological parameters of our model (1)–(4) are learnt by solving the optimzation problem of (5) to fit the clinical viral load data. The problem is solved by derivative free optimization (see Methods) and the results, plotted in Fig. 2 demonstrate a good match to the actual deceleration obtained by remdesivir. Since the clinical viral titre starts from a certain number of days post symptoms, this unknown incubation period is identified to be an average of $\varepsilon =4$ days. Note also that the count of immune T cells in the blood of the patients were not provided by^25^, but is predicted by the learnt model, which is significant given the paramount importance of the immune response attached to the pathogenesis of COVID-19^20^.

### Optimizing COVID-19 therapy

The timing of initiation of therapy represents a pivotal element in defining the therapeutic effects. The interval between the acquisition of the SARS-CoV-2 infection and treatment initiation will vary but, currently, the great majority of patients will be identified and receive treatment after the onset of symptoms and often when seriously ill^27^. Optimization of drug regimens requires a systems immunology approach that includes the pharmacokinetics of drug delivery and absorption, the pharmacodynamics of drug activity against the pathogen in the organism, the replication of the virus and the action of the immune cells.

The target disease free equilibrium can be found from

$$\begin{aligned}&\frac{EC_{50}}{D+EC_{50}}\mu V(1-\frac{V}{\sigma _v})-\beta VI-\alpha _vV =0 \nonumber \\&s_I+\delta I(\frac{V}{V+\sigma _i})-\alpha _iI =0 \nonumber \\&-\alpha _dD =0\;\; \tau _k<t<\tau _{k+1},\;\; k=1,2\ldots N \end{aligned}$$

which leads to the point $\{[V,I,D]\in \mathbb {R}^{3+}|V=0,I=\frac{s_i}{\alpha _i},D=0\}$, which also proves the homeostasis of immune T cells.

The Food and Drug Administration (FDA) and the World Health Organization (WHO) recommend treatment regimes for standard antiviral therapies and for repurposing them against COVID-19 for sympathetic use^28^. Therefore the baseline dose and schedule needs to be adapted and fine tuned so that the treatment is optimized for majority of patients coming in after the incubation period. The optimized pathological and pharmacological model developed thus far for antiviral treatment of COVID-19 on the basis of viral titre of hospitalized patients can be leveraged to develop a COVID-19 optimized model based antiviral drug schedule.

The multi objective control problem is to minimize the viral load as much as possible by administering minimal amount of drug in terms of dose and schedule, thereby minimizing the associated costs and unnecessary side effects. The optimization problem can then be stated as

6 $$\begin{aligned} \min _{\mathbf {D},N}\qquad&\Vert \mathbf {V}\Vert ^2 + \lambda \Vert \mathbf {D}\Vert ^2 \nonumber \\ \textit{where}\qquad&\dot{V} = \frac{EC_{50}}{D+EC_{50}}\mu V(1-\frac{V}{\sigma _v})-\beta VI-\alpha _vV \nonumber \\&\dot{I} = s_I+\delta I(\frac{V}{V+\sigma _i})-\alpha _iI \nonumber \\&\dot{D}=-\alpha _dD\;\; \tau _k<t<\tau _{k+1},\;\; k=1,2\ldots N \nonumber \\&0\le D\le D_s,\quad 1\le N \le N_s \end{aligned}$$

where the cost function now weighs between the vector of viral load $\mathbf {V}$ and the vector of drug administered $\mathbf {D}$ through a tunable parameter $\lambda$. The minimization is performed over the system wide pathological and pharmacological dynamic equality constraints as well as safety based inequality constraints that limit the maximum drug dose to $D_s$ and the length of the schedule to $N_s$ to be administered.

The pathogenic model developed thus far matched well with the viral titre and drug loading of^25^, albeit with possible side effects. Now considering the fact that the toxicities linked to long term treatment of microbial diseases are often less pronounced when used over a shorter time horizon for viral infections^9^ motivates us to explore an optimally reduced drug dosage and scheduling while ensuring an aggressive viral deceleration.

**Figure 3:**
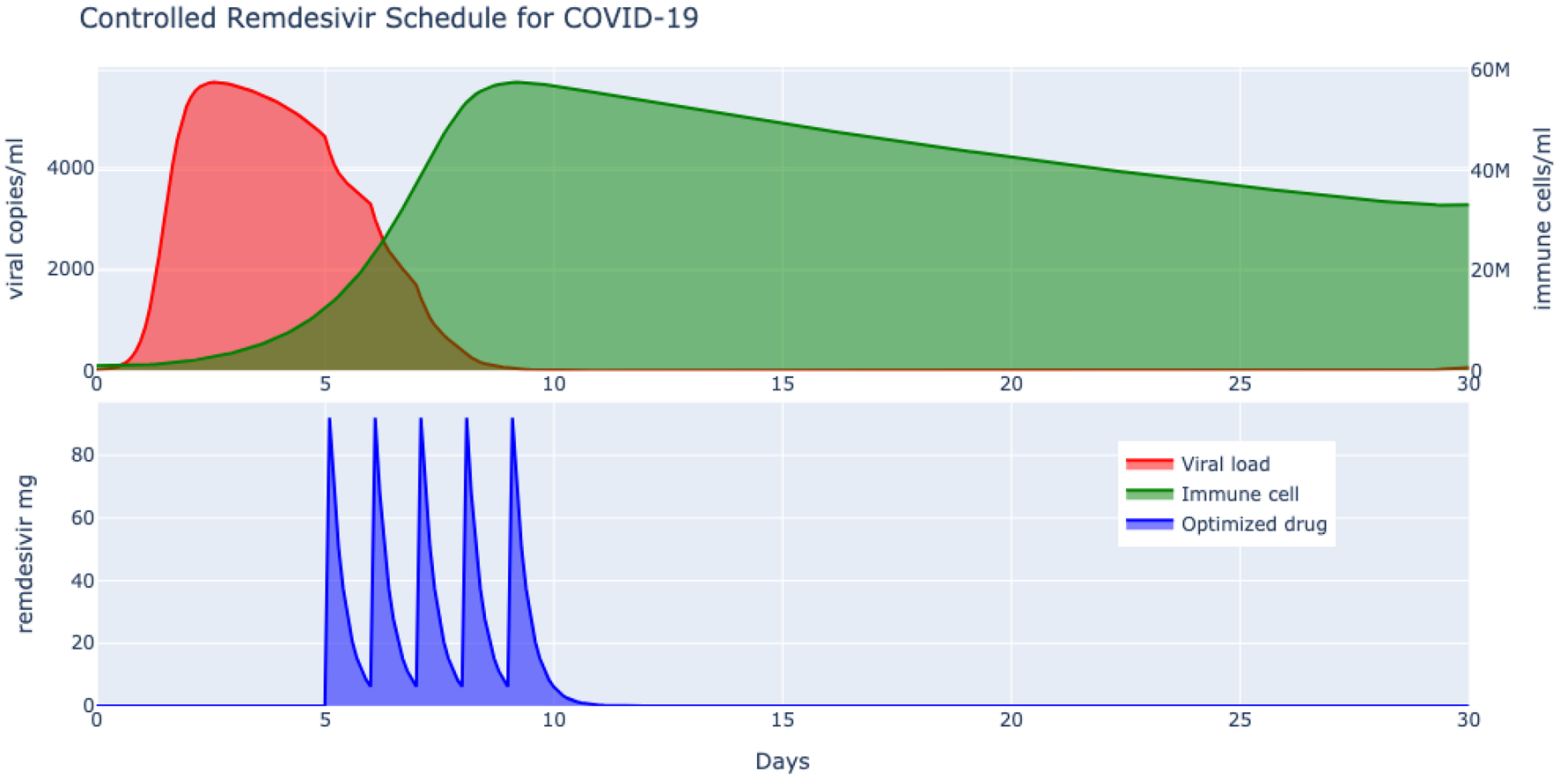
Optimized delivery of remdesivir results in much lower dosage and schedule with significant viral deceleration as compared to the prescribed higher dosage and schedule.

The controlled drug dosage and schedule is obtained by minimizing the cost function of (6) that penalizes higher drug dosage by a normalized factor of $\lambda =10^3$ over minimization of viral clearance. The safety constraint imposed on the drug loading is 200 mg on day 1 followed by a maintenance of 100 mg to a maximum of $N=10$ days. The minimization is solved by the derivative free stochastic global optimizer algorithm (see “Methods”) and the results are plotted in Fig. 3. What is immediately clear is the $50\%$ reduction in the drug scheduling to 5 days compared with a 10 day schedule of^25^ and also a $50\%$ reduction in the loading of remdesivir from 200 mg to about 90 mg. In order to compare any potential reduction in the treatment efficacy, the area under the curve of the optimized $V_a^*$ to the standard therapy $V_a$ can be represented as $100(1-\frac{V_a^*}{V_a})$^29^, which turns out to be $100(1-\frac{26612}{25408})\approx 0\%$ thereby confirming maintenance of an effective viral clearance achieved by a significant reduction in drug dose and schedule. The results obtained here, independently and analytically, are in fact supported by a number of clinical studies which advocate a 5 days remdesivir schedule^30^.

## Discussion

Given the continued urgency in demand for safe and effective therapeutics against COVID-19, remdesivir, a broad-spectrum antiviral drug emerged as a potential candidate. What was lacking was a systematic repurposing of remdesivir, originally used for Ebola to fight SARS-CoV-2. We filled in this gap by first identifying the pathogenesis of novel coronavirus from clinical data of patients acutely infected by COVID-19 and treated by remdesivir. This allowed us to further optimize the efficacy of remdesivir specific to the evolution of COVID-19 ensuring optimal viral deceleration with minimum drug administration. Since adverse effects of remdesivir have been detected and become a concern for clinicians^31^, the reduced antiviral drug dosing and scheduling obtained leading to a quantitative reduction in associated toxicities, is of paramount significance.

**Figure 4:**
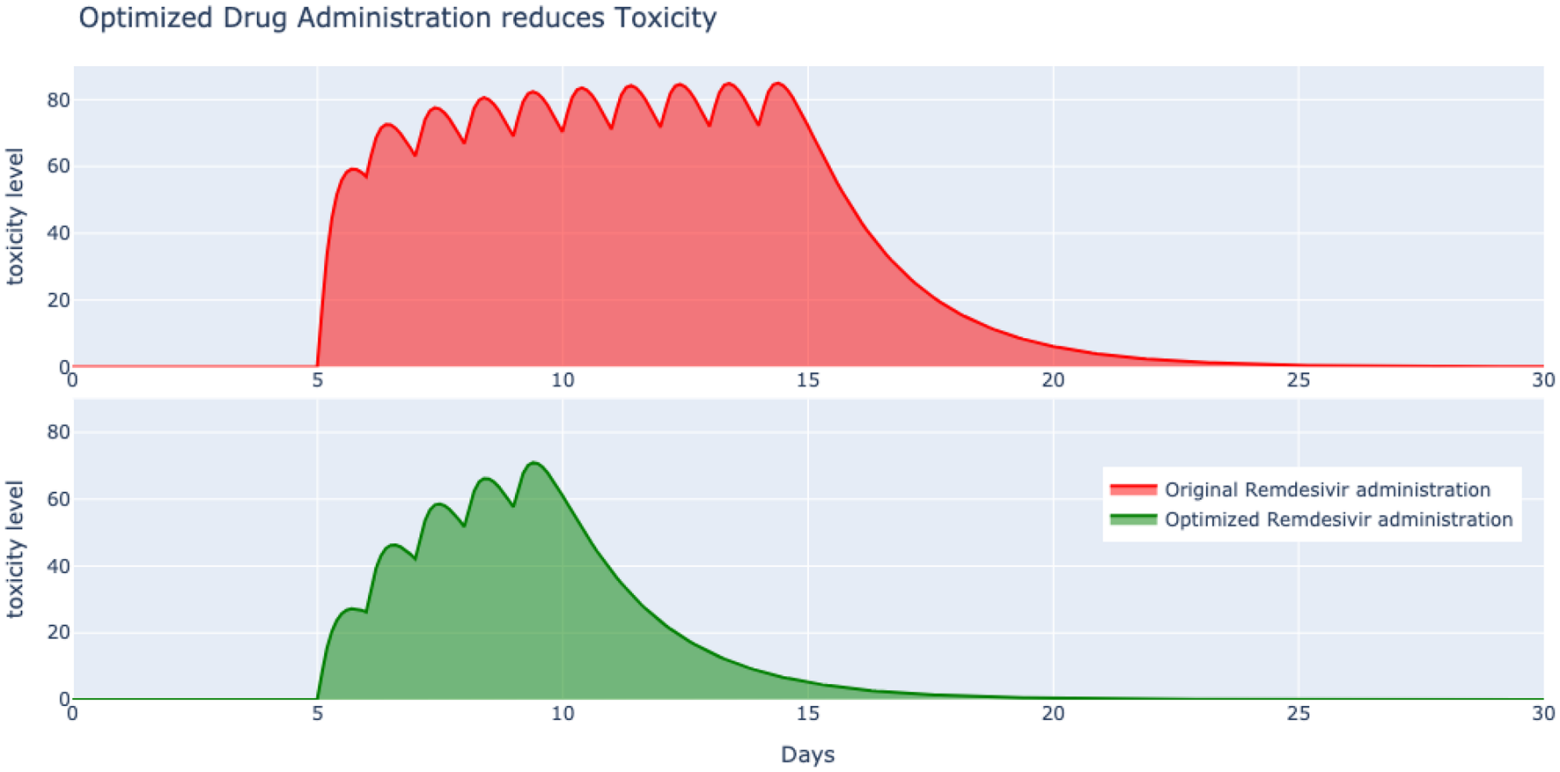
High toxicity is reported for the original remdesivir administration of 200 mg on day 1 followed by 100 mg/day from days 2 to 10 (top) compared to a much lower toxic level for the optimal repurposed remdesivir regimen of 90 mg/day from days 1 to 5 in treating COVID-19.

The overarching objective of nucleotide analogs like remdesivir is to stop the manufacture of new viral RNA, making it impossible for infected host cells to keep producing new virions. Nucleotide analogs can do this by embodying a base into the replicating strand from which viral RNA polymerase cannot elongate^32^. Neucleoside analogs are known to cause mitochondrial dysfunction by reducing sythesis of mitochondrial proteins and mitochondrial DNA. Sporadic cases of respiratory (distress), cardiovascular (hypotension) toxicity and nephrotoxicity (kidney injury), hepatotoxicity (elevated bilirubin, aminotransferase) and gastrointestinal symptoms (nausea and diarrhea)have been reported in the literature^31^.

The absence of concrete data on remdesivir toxicity^32^ and its current incomplete safety profile^31^, necessities the formulation of a toxicology model that can be appended to our pathology immunology pharmacology model. The relationship between the level of toxicity *T* inside the patient’s body and the drug concentration *D* is captured by

7 $$\begin{aligned} \dot{T} = D - \alpha _T T \end{aligned}$$

where parameter $\alpha _T$ indicates the rate of elimination of toxicity. This enables us to compute and compare the toxicity levels of the prescribed remdesivir administration of 200 mg on day 1 followed by 100 mg/day from days 2 to 10 with our optimized regimen of 90 mg/day from days 1 to 5 in treating COVID-19. The dramatic reduction in toxicity levels in case of the optimal antiviral therapy can be seen through the plots of Fig. 4. The reported toxicity levels are well in the range of $T\le 100$ as suggested by many researchers working on empirical data^33^. The quantitative reduction in toxicity can be computed as $100\cdot (1-\frac{T_a^*}{T_a})$, where $T_a^*, T_a$ are the area under the toxic level curves for the optimal and original schedules respectively. The toxicity reduction is $100\cdot (1-0.42)$ which is equal to $58\%$, a significant quantity.

This concurs with the reported findings that associated toxicities of remdesivir increase with elevated drug dosing^31,32^. Further, a study in Annals of Internal Medicine report that compared to a 10-day remdesivir course, a 5-day course may reduce time to recovery, mortality or serious adverse events among hospitalized patients and increase recovery or clinical improvement by moderate amounts^34^. Therefore, our pathological analysis and drug optimization grounded in systems theory forms the basis of the conclusions that a 5-day course of remdesivir can provide the same benefits with fewer harms and lower cost than a 10-day course^34^. It is also argued that longer treatment periods in patients can lead to development of resistance^35^, however there has been no significant evidence of remdesivir associated resistance arising in patients in clinical setting.

COVID-19 pandemic is the worst crisis of modern times that has ripped through the society with high fatality count, no treatment and lock down induced economic and mental turmoil. In a race to repurpose antiviral therapy, several clinical studies have been performed on the most promising drugs, however with no systems pharmacological methods of analysis thus far. This paper introduced systems and optimization based model learning of COVID-19 pathogenesis from clinical data, followed by an optimized drug schedule for remdesivir. The results could predict the dynamics response of immune T cells, incubation period and most notably a reduced drug dosage maintaining a high viral efficacy. By explicitly encoding safety and optimal dosing, controlled therapies result in much lower side effects and toxicities, thus elevating the quality of life.

The emergence of infections such as severe acute respiratory syndrome and COVID-19 have vividly demonstrated global vulnerability to infectious diseases and the need for robust health care systems to respond to such threats. The one-size-fits-all approach of repurposing existing antivirals to novel coronaviruses faces many hurdles such as patient approvals, extended clinical trials and associated high treatment costs. Due to limited availability, the remdesivir drug has been selling at very high inflated costs. The systematic analysis technique grounded in systems pharmacology that is developed and presented here can drastically cut the number of clinical trials and lead to tailored virus specific therapies. Additionally, the associated immune response prediction by our immunological interaction model informs the start of antiviral therapy, which is effective in the early stages and the need to curtail excessive immune responses by immunosuppresive agents like corticosteroids^36^.

Our technique makes it possible to learn the pathology immunology pharmacology model for any emerging infectious disease. Therefore, the framework is expected to translate to other deadly emerging infectious diseases due to urbanization such as Nipah virus and Bubonic plague. Moreover, due to the multi-objective nature of the model-based controlled intervention balancing efficacy with toxicity, many patients who are now excluded from antiviral therapy can be included thus widening the base. For instance, a pilot study presented in Infectious Diseases Society of America, administered the reduced 5-day course of remdesivir to patients with underlying liver cirrhosis and reported no acute elevations in aminotransferase levels or adverse events^37^. Our system theoretic pathological model based drug optimization is tailored to patient specific outcomes and can be delivered as personalized medicine. Accordingly, a significant positive impact on quality of life by advancement of systems medicine is expected.

## Methods

### Pathological parameter learning

The minimization problem of (5) is constrained to the dynamics of the nonlinear pathology pharmacology differential equations of (1)–(4), that cannot be integrated analytically, hence neither could the gradients of the cost function be computed. Therefore, a derivative free optimization technique of Nelder and Mead^38^ is adapted to solve the nonlinear optimization problem, the algorithm follows:

*Initialize*: An $n+1$ dimensional simplex is formed around the seed value of $p=[\mu ,\beta ,\sigma _v,\alpha _v,\delta ,\sigma _i,EC_{50},\varepsilon ]$, with associated cost *J*(*p*) from (5).
*Order*: Evaluate the cost function at vertices by numerically integration and sort $J(p_1)\le \ldots J(p_n)\le J(p_{n+1})$ and compute the centroid $p_0=\frac{1}{n}\sum _1^n p_i$.
*Terminate*: If standard deviation of simplex vertices is within specified tolerance, output optimal set of parameters as $p^*=p_1$.
*Reflect*: Compute $p_r = p_0 + \eta (p_0-p_{n+1})$ with $\eta >0$. If $J(p_1)\le J(p_r)<J(p_n)$, then $p_{n+1}=p_r$, go to Order.
*Expand*: If $J(p_r)<J(p_1)$, then $p_e=x_0+\mu (p_r-p_0)$. Further if $J(p_e)<J(p_r)$, then $p_{n+1}=p_e$ and go to Order, else $p_{n+1}=p_r$ and go to Order.
*Contract*: Here $J(p_r)\ge J(p_n)$, so $p_c=p_0+\rho (p_{n+1}-p_0)$. Further if $J(p_c)<J(p_{n+1})$, then $p_{n+1}=p_c$ and go to Order.
*Shrink*: Here $J(p_c)>J(p_{n+1})$, replace $p_i=p_1+\sigma (p_i-p_1)$ for all $i\in [2,n+1]$ and go to Order.

The algorithm terminates with the optimized values of the pathological and pharmacological parameters *p* that justify the recorded viral titre.

### Optimizing antiviral therapy

The optimization problem of (6) is nonlinear due to the integration of the underlying pathological differential equations (1)–(4). The cost function can neither be differentiated analytically due to the underlying coupled nonlinear differential equations that need numerical integration. Also, note the addition of inequality constraints further complicates the problem. Hence, a derivative free constrained stochastic global optimization technique based on differential evolution^39^ is adapted to solve the multi-objective constrained minimization of (6), the algorithm follows

*Define*: The cost function $f(x):X\subseteq \mathbb {R}^P\longrightarrow \mathbb {R}$ of (6) is to be minimized over parameters $x=\mathbf {D},N$ with the optimal parameter $x^*\in X$ such that $f(x^*)\le f(x),\;\forall x\in X$. Pick a population size of at least $N\ge 4$ and define the parameter vector as $x_{i,G}=[x_{1,i,G},x_{2,i,G},\ldots x_{P,i,G}]$ with $i=1,2,\ldots N$.
*Initialize*: Set the bounds on the parameters (drug dosage and schedule): $x_j^L\le x_{j,i,1}\le x_j^U$ and randomly select initial parameter values uniformly on the intervals $[x_j^L,x_j^U]$.
*Mutate*: For a given target vector $x_{i,G}$, randomly select 3 vectors $x_{r1,G},x_{r2,G},x_{r3,G}$ such that *i*, *r*1, *r*2, *r*3 are distinct. Form the donor vector $y_{r1,G+1}=x_{r1,G}+F(x_{r2,G}-x_{r3,G})$ with the mutation factor $F\in [0,2]$.
*Recombine*: Form the trial vector $\forall i=1,2,\ldots N$ and $\forall j=1,2,\ldots P$ $$z_{j,i,G+1}=\bigg \{\begin{matrix}y_{j,i,G+1}&{} if&{} rand_{ji}\le CR&{} or&{} j=I_{rand} \\ x_{j,i,G} &{} if&{} rand_{ji}>CR&{} and&{} j\ne I_{rand}\end{matrix}$$. The crossover probability $CR\in [0,1]$ and $I_{rand}$ ensures $y_{i,G+1}\ne x_{i,G}$.
*Select*: Admit vectors with lowest function values to next generation $$x_{i,G+1}=\bigg \{\begin{matrix}z_{i,G+1}&{} if&{} f(z_{i,G+1})\le f(x_{i,G}) \\ x_{i,G} &{} otherwise\end{matrix}$$ for all $i=1,2,\ldots N$.
*Terminate*: Check termination criteria in terms of cost decrement or number of iterations. If satisfied, return the agent $x^*$ from the population with the least cost $f(x^*)$, otherwise go to *Mutate*.

The algorithm outputs the optimal set of drug doses and schedule *x*, subject to safety constraints that balances the multi-objective cost in terms of decelerating the viral load with minimal therapeutic interventions.

## Data Availability

All data generated in this study are have been included in this paper.
